# Immune Profile of Obese People and In Vitro Effects of Red Grape Polyphenols on Peripheral Blood Mononuclear Cells

**DOI:** 10.1155/2017/9210862

**Published:** 2017-01-24

**Authors:** Thea Magrone, Emilio Jirillo, Anna Spagnoletta, Manrico Magrone, Matteo Antonio Russo, Sergio Fontana, Flavia Laforgia, Ilaria Donvito, Angelo Campanella, Franco Silvestris, Giovanni De Pergola

**Affiliations:** ^1^Department of Basic Medical Sciences, Neuroscience and Sensory Organs, University of Bari, 70124 Bari, Italy; ^2^Fondazione San Raffaele, 72013 Ceglie Messapica, Italy; ^3^Consorzio MEBIC, San Raffaele University, Rome, Italy; ^4^Farmalabor Srl, 76012 Canosa di Puglia, Italy; ^5^Department of Biomedical Sciences and Human Oncology, Section of Clinical Oncology, University of Bari, 70124 Bari, Italy

## Abstract

The in vitro ability of polyphenols, extracted from red grape, to modulate peripheral blood mononuclear cell responses has been evaluated in 20 obese (Ob) people. With regard to cytokine release in response to phorbol myristate acetate (PMA), levels of interleukin-2 (IL-2), interferon-*γ* (IFN-*γ*), IL-4, IL-10, and IL-17 were higher in the Ob than in healthy (H) subjects.* Vice versa*, IL-21 concentrations were detected only in H people but they were undetectable in the Ob counterpart. In general terms, levels of IL-1*β*, IL-6, IL-8, and tumor necrosis factor-*α* were higher in Ob people when compared to H controls. On the other hand, polyphenols did not modify IFN-*γ*, IL-4, and IL-17 levels. However, an increase in IL-2 was observed in H individuals, whereas its levels were decreased in the Ob counterpart. Polyphenols significantly increased IL-10 release from H donors, whereas a trend to increase was observed in Ob people. In addition, polyphenols were able to significantly increase levels of H IL-21, while this was not the case in Ob people. Since IL-21 is an inducer of Th17 cells, it is likely that polyphenols may suppress the sources of this cytokine* via* production of IL-10. Accordingly, polyphenols decreased IL-1*β* and IL-6 release in comparison to H controls.

## 1. Introduction

Obesity is a worldwide epidemic in continuous growth, which is associated to high mortality and morbidity [1, 2]. It is characterized by an early inflammation of adipose tissue, which later on becomes systemic, thus resulting in type 2 diabetes, cardiovascular disease, and cancer [3–6]. From an immunological point of view, obesity is featured by several perturbations of immune response [7, 8]. Furthermore, immune cells and adipocytes both represent a remarkable source of inflammatory cytokines in the context of visceral fat [9]. Several immune cells, even including T cells, B cells, macrophages, and neutrophils have been identified in adipose tissue, and obesity seems to influence both quantity and profile of immune cell subsets. For these reasons, adipose tissue has been considered as a large immunologically and endocrinologically active organ in the course of obesity [10, 11]. However, despite the presence of hematopoietic lineage cells in adipose tissue, their direct role in immune surveillance or host defense needs further investigation [10]. Pandolfi et al. [12] have provided recent evidence for the high inflammatory role exerted by T helper (h) 17 cells in visceral adipose tissue from obese (Ob) people, with release of elevated levels of interleukin (IL)-1*β*, IL-6, and IL-17. At the same time, production of IL-21 contributes to perpetuate inflammation* via* activation of another wave of Th17 cells [6, 13]. It is to note that expansion of Th17 cells in visceral fat is associated to a decrease of Treg cells [9, 11].

In Ob individuals, the number of leukocytes in the blood is increased as compared to the lean counterpart [14]. In particular, monocyte number is elevated and reduction of fat body positively correlates to the decrease of these cells [8, 15, 16]. Then, monocytes migrate to adipose tissue and differentiate into M1 and M2 macrophages [17]. The former play an inflammatory function and are predominant in Ob adipose tissue, while the latter are more prevalent in lean adipose tissue, with production of the anti-inflammatory cytokine IL-10 [18]. In terms of adaptive immunity, exaggerated release of leptin (an adipokine) from adipocytes of the visceral fat acts upon Th1 and Th2 cells, thus increasing production of IL-2 and IL-4 as well as T lymphocyte proliferation [19, 20]. The activity of leptin is reinforced by the contemporary release of resistin and visfatin, which both contribute to the production of IL-1*β*, IL-6, IL-8, and IL-12, respectively [19, 20].

Polyphenols are natural compounds largely present in fruits, vegetables, and cereals. They are endowed with antioxidant and anti-inflammatory activities, as shown in animal models and clinical trials [21]. According to our personal experience, using polyphenols, isolated from red wine and fermented grape marc (FGM), we have found that these compounds are able to induce and activate peripheral human Treg cells [22]. In turn, release of IL-10 by these cells led to a condition of immune homeostasis among the various T cell subsets [23]. Also a direct effect of FGM on human polymorphonuclear cells, monocytes, and basophils was observed in terms of reduced release of reactive oxygen species (ROS) and inhibition of basophil degranulation, respectively [24]. Furthermore, in vitro treatment of human monocytes by red wine polyphenols inhibited binding of endotoxins to Toll-like receptor 4, with reduced activation of NF-*κ*B and decreased release of proinflammatory cytokines [25].

In obesity, the beneficial effects of dietary polyphenols have been reported by several authors. Ricordi et al. [26] have pointed out the ability of polyphenols to exert an anti-inflammatory activity in obesity, diabetes, and metabolic syndrome, thus leading to the modulation of glucose and insulin levels. Ríos-Hoyo et al. [27] have shown the properties of polyphenols to avoid cardiovascular complications of obesity, thus promoting vasodilation, antiatherogenetic, and antithrombotic effects. Moreno-Indias et al. [28] have reported the effects of a moderate intake of red wine polyphenols on the modulation of the gut microbiota and reduction of inflammatory markers in Ob people. Experimentally, green tea polyphenols have been shown to attenuate inflammatory functions of Ob rat neutrophils in terms of decreased release of ROS and proinflammatory cytokines [29]. Lastly, Molina et al. [30] have documented in Ob rats, treated with green tea polyphenols, a reduced release of inflammatory cytokines and an increase in IL-10.

On these bases, the aim of the present work is to investigate the in vitro effects of polyphenols isolated from seeds of red grape (Nero di Troia cultivar) on peripheral blood mononuclear cells (PBMCs) from Ob people. Both absolute numbers of PBMCs and cytokine release (Th1-, Th2- Treg-, Th17-related cytokines and canonical proinflammatory cytokines) will be evaluated in the presence or the absence of polyphenols in comparison to healthy (H) donors.

## 2. Materials and Methods

### 2.1. Enrolment of Ob People

Ob individuals were consecutively enrolled at the outpatient ambulatory of the Clinical Oncology Section, c/o Department of Biomedical Sciences and Human Oncology, University of Bari, School of Medicine, Bari, Italy. They were addressed by family doctors to the Outpatient Clinic for losing weight and receiving dietary and lifestyle recommendations.

With regard to the criteria of inclusion to the study, subjects having a body mass index (BMI) over 25.0, older than 18 years, and not using any kind of drug (including hormone replacement therapy and oral contraception for pre- and postmenopausal manifestations, resp.) were enrolled. Actually, at the moment of the recruitment, they were still untreated for their condition of obesity.

Exclusion criteria were represented by having diabetes mellitus, chronic inflammatory diseases, and cardiovascular disease. On these bases, according to the inclusion and exclusion criteria, twenty consecutive Ob individuals were enrolled (eight males and twelve females).

Informed consent was verbally given by all subjects recruited, and approval of the study was in accordance with the ethical standards of the ad hoc local committee on human experimentation as well as with the 1975 Helsinki declaration, as revised in 2000.

No participant was on a limited calorie diet nor had been taking part in intense physical activity before enrollment. The day prior to immunological evaluation, the subjects were requested to refrain from taking alcohol and caffeine.

### 2.2. Normal Donors

Twenty heparinised peripheral blood samples from H donors (age range: 32–52) were recruited at the Blood Bank from the Polyclinic Hospital, Bari, Italy, after giving a written consent.

### 2.3. Polyphenol Extraction

*Canosina* red grape from Nero di Troia is an autochthonous* Vitis vinifera* grape cultivar which grows in Apulia (South Italy). It is characterized by thick skinned and small sized berries. Frozen seeds from berries were extracted by percolation using ethanol/water (70 : 30). Then, the extract was first analyzed by means of liquid chromatography with diode array detection to define the polyphenol composition. Thereafter, the extract was purified on a synthetic adsorbent brominated resin and percentage of polyphenol content was determined. The extracts were evaluated for their potential antioxidant effects by using the 2,2-diphenyl-1-picrylhydrazyl assay which measures the ability of test agents to scavenge radicals [31].

### 2.4. Experimental Design

PBMCs from both H and Ob people were separated on a gradient of Ficoll and resuspended in complete medium constituted by RMPI 1640 (Miltenyi Biotec, Bergisch Gladbach, GE) and supplemented with gentamycin (Sigma-Aldrich, Milan, Italy), penicillin (100 mg/mL) (Biowhittaker, Walkersville, USA), sodium pyruvate (Sigma-Aldrich), hepes buffer (Sigma-Aldrich), and 30% fetal bovine serum (Biowhittaker). PBMCs were adjusted to a concentration of 3 × 10^6^/ml and, then, stimulated with three different amounts (1, 3, and 5 *μ*g, resp.) of polyphenols. These PBMC samples were used either to characterize their absolute numbers or to evaluate the release of various cytokines by means of a flow cytometric assay. Unstimulated PBMCs served as negative controls, while cells treated with phorbol myristate acetate (PMA) (Sigma-Aldrich) plus ionomicyn (Sigma-Aldrich) served as positive controls from both H and Ob individuals. The different cell suspensions were separately incubated for 24 h, at 37°C, 5%/CO_2_. Then, supernatants of each sample were collected and centrifuged at 10000 rpm for 10 min and stored at −30°C until use. Instead, cell pellets of each sample were utilized to characterize the absolute numbers of PBMCs.

### 2.5. Analysis of Absolute Numbers of PBMCs

Cell suspension were dispensed into two different tubes and, then, separately added with the following monoclonal antibodies: CD3FITC, CD8PE, CD4APC, CD45APC-Cy7, CD3FITC, CD16PECD56PE, and CD19APC, respectively.

After 40 min incubation at 37°C in the dark, each tube was centrifuged at 1200 rpm for 10 minutes. Thereafter, supernatants were discarded and each tube was added with 500 *μ*l of phosphate buffer saline (PBS).

Marked cells were then analyzed by flow cytometry [FACS Aria III, Becton Dickinson (BD), Milan, Italy].

Data acquisition and analysis were performed with the FACS Diva Software version 6.1.3 in the form of list-mode data files version FCS 3.00, using the default biexponential transformation.

### 2.6. Cytokine Assay

Cell supernatants were incubated with the cytometric bead array (CBA) human soluble protein Flex Set kit (BD). This kit permitted to determine the following cytokines: IL-2; IL-12; IFN-*γ*; IL-4; IL-10; IL-17; IL-21; IL-1*β*; IL-6; IL-8 and tumor necrosis factor (TNF)-*α*.

The capture beads provided in each CBA human soluble protein Flex Set were dispensed in each cell supernatant and, after 1 h incubation at dark, a human soluble protein Flex Set PE detection was added for 2 h in the dark. Afterwards, each sample suspended in 1 ml PBS was centrifuged at 800 rpm 10 min, then supernatants were carefully discarded, and finally, 500 *μ*l PBS was added to pellets.

Samples were acquired using the FACS DIVA software, which is able to distinguish different types of cytokines according to some properties such as forward scatter, side scatter, and mean fluorescence of the following fluorochromes: PE, APC e APC-Cy7. The standard curve for all cytokines was initially prepared. The FCAP software (BD) was used to quantify all cytokines in standards and samples.

### 2.7. Statistical Analyses

Statistical analysis was performed using Bonferroni's test for comparison between H and Ob samples. GraphPad Prism statistical software release 5.0 for Windows Vista was used. Statistical significance was set at *p* < 0.05.

## 3. Results

In Table 1, demographic and anthropometric features of Ob individuals recruited are illustrated.

### 3.1. Absolute Numbers of PBMCs

**Figure 1:**
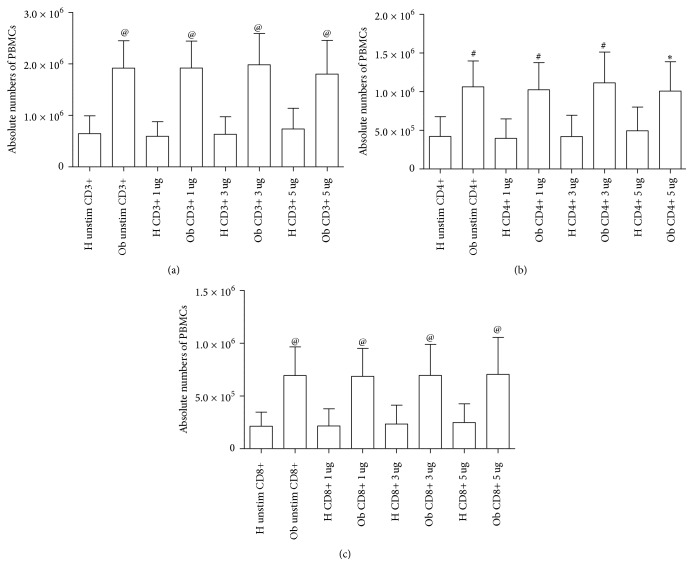
Absolute numbers of PBMCs from H and Ob individuals. (a) Absolute numbers of CD3+ cells from untreated and treated cells with 1, 3, and 5 *μ*g polyphenols, respectively. (b) Absolute numbers of CD4+ cells from unstimulated and treated cells with 1, 3, and 5 *μ*g dose polyphenols, respectively. (c) Absolute numbers of CD8+ cells from untreated and treated cells with 1, 3, and 5 *μ*g dose polyphenols, respectively. H = healthy donors, Ob = obese people. Statistical analysis was performed using the GraphPad Prism statistical software release 5.0 for Windows Vista. Bonferroni's test was used for comparison between the H and Ob samples. Statistical significance was set at *p* < 0.05. (a) ^@^*p* < 0.0001 H unstimulated* versus* Ob unstimulated, ^@^*p* < 0.0001 H 1 *μ*g/ml* versus* Ob 1 *μ*g/ml, ^@^*p* < 0.0001 H 3 *μ*g/ml* versus* Ob 3 *μ*g/ml; ^@^*p* < 0.0001 H 5 *μ*g/ml* versus* Ob 5 *μ*g/ml: (b) ^#^*p* < 0.001 H unstimulated* versus* Ob unstimulated, ^#^*p* < 0.001 H 1 *μ*g/ml* versus* Ob 1 *μ*g/ml, ^#^*p* < 0.001 H 3 *μ*g/ml* versus* Ob 3 *μ*g/ml, ^*∗*^*p* < 0.05 H 5 *μ*g/ml* versus* Ob 5 *μ*g/ml; (c) ^@^*p* < 0.0001 H unstimulated* versus* Ob unstimulated, ^@^*p* < 0.0001 H 1 *μ*g/ml* versus* Ob 1 *μ*g/ml, ^@^*p* < 0.0001 H 3 *μ*g/ml* versus* Ob 3 *μ*g/ml, ^@^*p* < 0.0001 H 5 *μ*g/ml* versus* Ob 5 *μ*g/ml.

**Figure 2:**
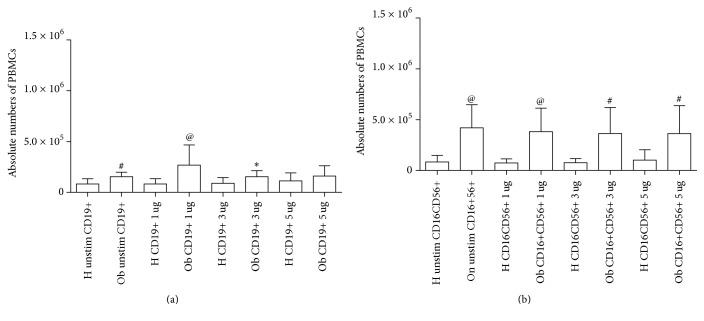
Absolute numbers of PBMCs in H and Ob individuals. (a) Absolute numbers of CD19+ cells from untreated and treated cells with 1, 3, and 5 *μ*g dose polyphenols, respectively. (b) Absolute numbers of CD16+CD56+ cells from untreated and treated cells with 1, 3, and 5 *μ*g dose polyphenols, respectively. For abbreviations and statistical analysis, see Figure 1. (a) ^#^*p* < 0.001 H unstimulated* versus* Ob unstimulated, ^@^*p* < 0.0001 H 1 *μ*g/ml* versus* Ob 1 *μ*g/ml, ^*∗*^*p* < 0.05 H 3 *μ*g/ml* versus* Ob 3 *μ*g/ml (b) ^@^*p* < 0.0001 H unstimulated* versus* Ob unstimulated, ^@^*p* < 0.0001 H 1 *μ*g/ml* versus* Ob 1 *μ*g/ml, ^#^*p* < 0.001 H 3 *μ*g/ml* versus* Ob 3 *μ*g/ml, ^#^*p* < 0.001 H 5 *μ*g/ml* versus* Ob 5 *μ*g/ml.

In Figures 1(a)–1(c) and in Figures 2(a) and 2(b), absolute numbers of PBMCs, as determined by flow cytometry, are indicated. The cell absolute numbers of CD3+ (Figure 1(a)) CD4+ (Figure 1(b)), CD8+ (Figure 1(c)), CD19+ (Figure 2(a)), and CD16+CD56+ (Figure 2(b)) were significantly higher in Ob people than in the H group.

In both figures, the effects of polyphenols on the absolute numbers of PBMCs are indicated. These data will be discussed in a specific subheading in this section.

### 3.2. Effects of PMA on Th1 and Th2-Related Cytokine Release

**Figure 3:**
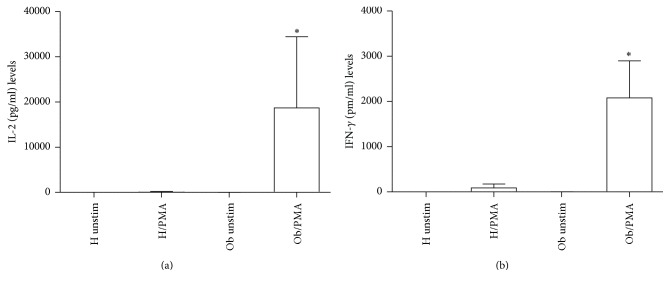
Release of Th1-related cytokines in presence or absence of PMA. (a) IL-2 levels released from H and Ob PBMCs in presence or absence of PMA. (b) IFN-*γ* levels released from H and Ob PBMCs in presence or absence of PMA. For abbreviations and statistical analysis, see Figure 1. (a) ^*∗*^*p* < 0.0001 H/PMA* versus* Ob/PMA; ^*∗*^*p* < 0.0001 Ob unstimulated* versus* Ob/PMA; (b) ^*∗*^*p* < 0.0001 H/PMA* versus* Ob/PMA; ^*∗*^*p* < 0.0001 Ob unstimulated* versus* Ob/PMA.

**Figure 4:**
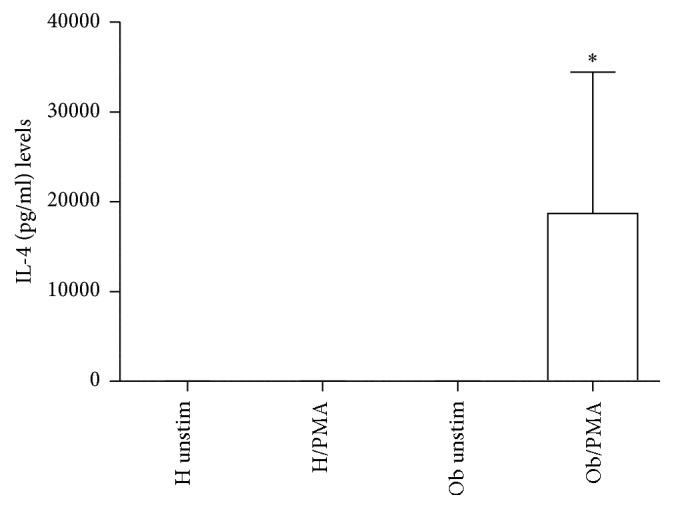
Release of Th2-related cytokine in presence or absence of PMA. IL-4 levels released from H and Ob PBMCs in presence or absence of PMA. For abbreviations and statistical analysis, see Figure 1. ^*∗*^*p* < 0.0001 H/PMA* versus* Ob/PMA: ^*∗*^*p* < 0.0001 Ob unstimulated* versus* Ob/PMA.

In Figure 3, release of IL-2 (a) and IFN-*γ* (b) is represented. Both Th1-related cytokine spontaneous release is undetectable in H and Ob individuals, respectively. However, in the presence of PMA, only Ob subjects produced elevated amount of IL-2 and IFN-*γ*. In Figure 4, IL-4 production increased in a statistically significant manner only in Ob PBMC in presence of PMA.

### 3.3. Effects of PMA on Treg/Th17-Related Cytokines

**Figure 5:**
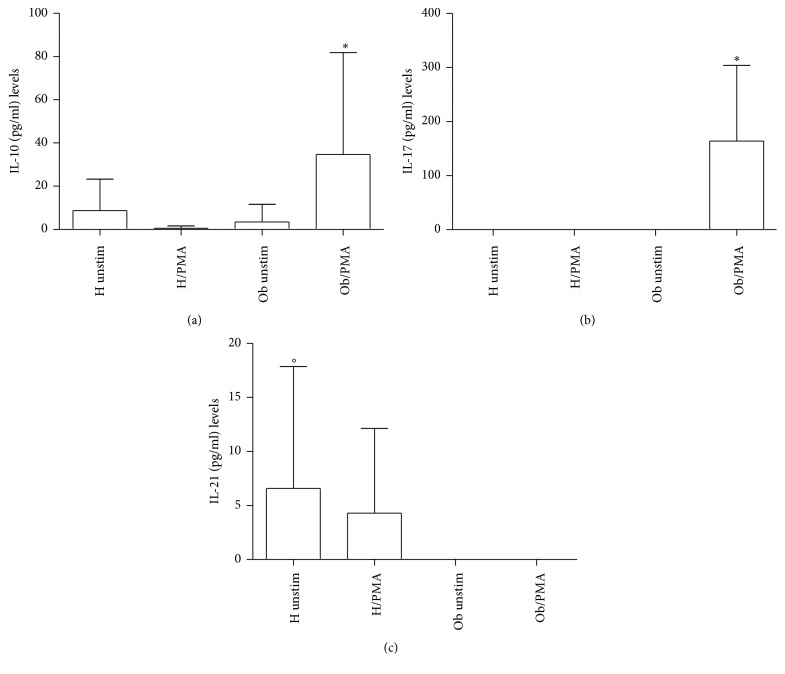
Release of Treg/Th17-related cytokines. (a) IL-10 levels released from H and Ob PBMCs in presence or absence of PMA. (b) IL-17 levels released from H and Ob PBMCs in presence or absence of PMA. (c) IL-21 levels released from H and Ob PBMCs in presence or absence of PMA. For abbreviations and statistical analysis, see Figure 1. (a) ^*∗*^*p* < 0.0001 H/PMA* versus* Ob/PMA; ^*∗*^*p* < 0.0001 Ob unstimulated* versus* Ob/PMA; (b) ^*∗*^*p* < 0.0001 H/PMA* versus* Ob/PMA, ^*∗*^*p* < 0.0001 Ob unstimulated* versus* Ob/PMA; (c) °*p* < 0.05 H unstimulated* versus* Ob unstimulated.

In Figure 5(a), levels of IL-10 significantly increased in presence of PMA in Ob PBMCs only. Conversely, H and Ob untreated PBMCs did not release significant amounts of IL-10. Detectable levels of IL-17 (Figure 5(b)) were documented only in the case of PMA-treated Ob PBMCs. Instead, IL-21 release (Figure 5(c)) was observed only in unstimulated and PMA-stimulated H PBMCs in comparison with the Ob counterpart where levels of this cytokine were undetectable.

### 3.4. Effects of PMA on Proinflammatory Cytokine Release

**Figure 6:**
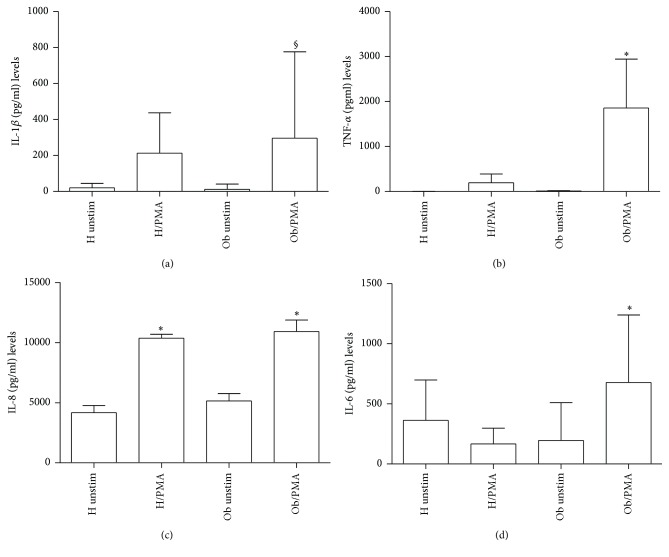
Release of proinflammatory cytokines. (a) IL-1*β* levels released from H and Ob PBMCs in presence or absence of PMA. (b) TNF-*α* levels released from H and Ob PBMCs in presence or absence of PMA. (c) IL-8 levels released from H and Ob PBMCs in presence or absence of PMA. (d) IL-6 levels released from H and Ob PBMCs in presence or absence of PMA. For abbreviations and statistical analysis, see Figure 1. (a) ^§^*p* < 0.001 Ob unstimulated* versus* Ob/PMA; (b) ^*∗*^*p* < 0.0001 H/PMA* versus* Ob/PMA, ^*∗*^*p* < 0.0001 Ob unstimulated* versus* Ob/PMA; (c) ^*∗*^*p* < 0.0001 H unstimulated* versus* H/PMA, ^*∗*^*p* < 0.0001 Ob unstimulated* versus* Ob/PMA; (d) ^*∗*^*p* < 0.0001 H/PMA* versus* Ob/PMA, ^*∗*^*p* < 0.0001 Ob unstimulated* versus* Ob/PMA.

In Figure 6(a), IL-1*β* was significantly increased in Ob PBMCs in presence of PMA in comparison to Ob untreated cells. In H individuals levels of this cytokine were lower than the Ob counterpart. TNF-*α* amounts (Figure 6(b)) were statistically significant in PMA-treated Ob PBMCs in comparison to Ob untreated and PMA-treated H PBMCs, respectively. In Figure 6(c), there is evidence that all samples produced IL-8, whose levels were statistically significantly higher in both Ob and H cells treated with PMA in comparison with untreated PMBCs, respectively. IL-6 levels (Figure 6(d)) were statistically significantly higher in Ob cells plus PMA in comparison with untreated Ob and H cells plus PMA, respectively.

### 3.5. In Vitro Effects of Polyphenols on H and Ob PBMCs

Polyphenols did not modify absolute numbers of H and Ob PBMCs at all concentrations, as indicated in Figures 1 and 2.

**Figure 7:**
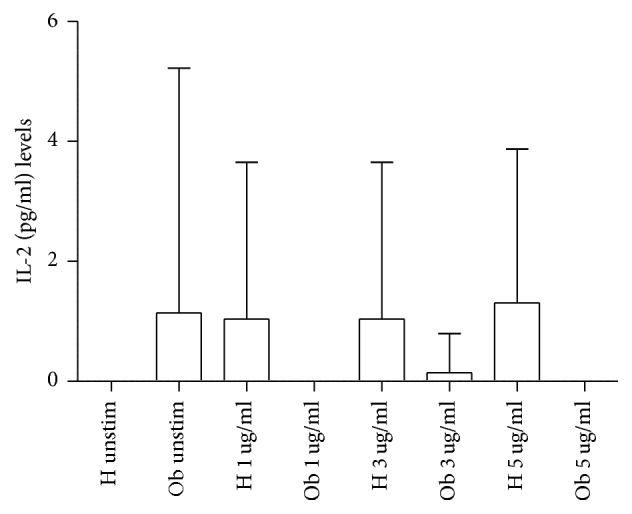
Release of Th1-related cytokines in presence or absence of polyphenols. IL-2 levels released from H and Ob PBMCs in presence or absence of 1, 3, and 5 *μ*g dose polyphenols, respectively. For abbreviations, see Figure 1.

Moreover, treatment with polyphenols did not affect the release of IFN-*γ* and IL-4 (data not shown). Conversely, as indicated in Figure 7, while polyphenols moderately increased IL-2 release from H PBMCs, they decreased the spontaneous production of this cytokine from Ob PBMCs.

**Figure 8:**
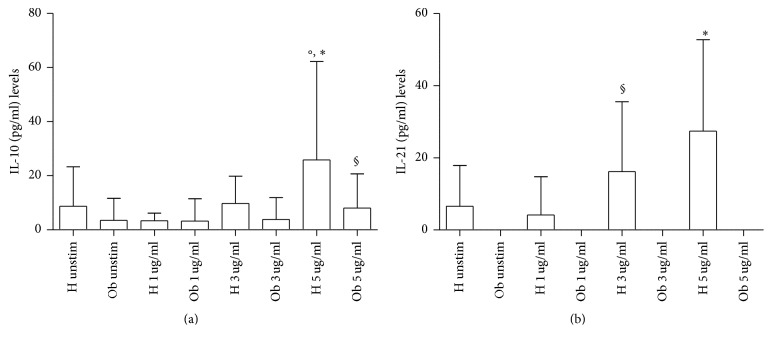
Release of Treg related cytokines in presence or absence of polyphenols. (a) IL-10 levels released from H and Ob PBMCs in presence or absence of 1, 3, and 5 *μ*g dose polyphenols, respectively. (b) IL-21 levels released from H and Ob PBMCs in presence or absence of 1, 3, and 5 *μ*g dose polyphenols, respectively. For abbreviations and statistical analysis, see Figure 1. (a) °*p* < 0.05 H unstimulated* versus* H 5 *μ*g/ml, ^*∗*^*p* < 0.0001 H 1 *μ*g/ml* versus* H 5 *μ*g/ml, °*p* < 0.05 H 3 *μ*g/ml* versus* H 5 *μ*g/ml, ^§^*p* < 0.001 H 5 *μ*g/ml* versus* Ob 5 *μ*g/ml; (b) ^*∗*^*p* < 0.0001 H unstimulated* versus* H 5 *μ*g/ml, ^*∗*^*p* < 0.0001 H 1 *μ*g/ml* versus* H 5 *μ*g/ml, ^§^*p* < 0.001 H 3 *μ*g/ml* versus* Ob 3 *μ*g/ml, ^*∗*^*p* < 0.0001 H 5 *μ*g/ml* versus* Ob 5 *μ*g/ml.

In Figure 8(a), IL-10 release from H and Ob PBMCs is expressed. Quite interestingly, IL-10 production from H cells was increased in the presence of 5 *μ*g polyphenols in comparison to unstimulated cells. Of note, with the same dose of polyphenols, a trend to increase of IL-10 levels from Ob PBMCs in comparison with unstimulated cells was observed.

In Figure 8(b), IL-21 release from H and Ob PBMCs is depicted. Quite interestingly, no release of IL-21 from unstimulated and polyphenol-stimulated Ob cells was noted. Conversely, polyphenols at all doses were able to significantly increase the release of IL-21 from H cells.

IL-17 was not detectable in H and Ob PBMCs, respectively, in the presence of polyphenols (data not shown).

**Figure 9:**
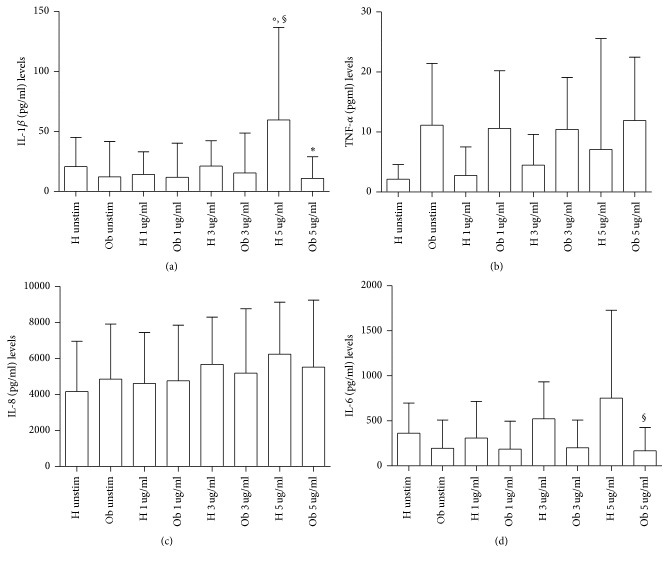
Release of proinflammatory cytokines in presence or absence of polyphenols. (a) IL-1*β* levels released from H and Ob PBMCs in presence or absence of 1, 3, and 5 *μ*g dose polyphenols, respectively. (b) TNF-*α* levels released from H and Ob PBMCs in presence or absence of 1, 3, and 5 *μ*g dose polyphenols, respectively. (c) IL-8 levels released from H and Ob PBMCs in presence or absence of 1, 3, and 5 *μ*g dose polyphenols, respectively. (d) IL-6 levels released from H and Ob PBMCs in presence or absence of 1, 3, and 5 *μ*g dose polyphenols, respectively. For abbreviations and statistical analysis, see Figure 1. (a) °*p* < 0.05 H unstimulated* versus* H 5 *μ*g/ml, ^§^*p* < 0.001 H 1 *μ*g/ml* versus* H 5 *μ*g/ml, °*p* < 0.05 H 3 *μ*g/ml* versus* H 5 *μ*g/ml, ^*∗*^*p* < 0.0001 H 5 *μ*g/ml* versus* Ob 5 *μ*g/ml; (d) ^§^*p* < 0.001 H 5 *μ*g/ml* versus* Ob 5 *μ*g/ml.

In Figure 9, release of IL-1*β* (a), TNF-*α* (b), IL-8 (c), and IL-6 (d), respectively, is expressed. As far as IL-1*β* release is concerned, 5 *μ*g polyphenols increased its release in H cells. Polyphenols were not effective at all doses on Ob cells. Instead, TNF-*α* and IL-8 were produced by H and Ob cells in the presence of polyphenols at the same manner without any statistically significant difference. With regard to Ob IL-6, a decrease of this cytokine in the presence of 5 *μ*g polyphenols was observed in comparison to the H counterpart.

## 4. Discussion

Obesity is a serious health problem worldwide, characterized by a systemic inflammatory status and comorbidities, such as type II diabetes, cardiovascular disease, and cancer [3–7, 32]. Our present data confirm and extend the reported altered immunological homeostasis in obesity [8, 14–16]. Accordingly, our evidence for the increased absolute numbers of Ob PBMCs seems to be related to the exaggerated secretion of IL-2 in response to PMA stimulation of Ob cells. Quite interestingly, H PBMCs are unresponsive to PMA and their absolute numbers are lower than those of Ob stimulated peripheral immune cells. These data suggest a condition of preexisting activation of Ob Th1 cells, the major source of IL-2, which acts as a potent growth factor [33]. In this direction, in vivo several circulating antigens, such as saturated fatty acids and bacterial endotoxins, continuously stimulate Ob immune cells, thus leading to their hyperactivation with a consequent imbalance of the immune networks [34, 35]. At the same time, the observed augmented release of IFN-*γ* by PMA-stimulated Ob PBMCs may contribute to the maintenance of the inflammatory status with release of other waves of proinflammatory cytokines and ROS from monocytes-macrophages and neutrophils [36]. Conversely, in our test system, H PBMCs do not produce both IL-2 and IFN-*γ* in response to PMA stimulation. Similar pattern of response can be observed in the case of the increased secretion of IL-4 by PMA-stimulated Ob PBMCs. Also in this instance, H Th2 cells do not produce IL-4 under the same experimental conditions. Increased amounts of IL-4 in obesity may contribute to a more sustained production of antibodies, involved in the formation of immune complexes, and/or auto antibodies [37]. In addition, the frequent association of asthma with obesity may rely on a more pronounced IgE switch in response to environmental allergens [38]. Our data on the Treg/Th17-related cytokines provide new insights on the immune pathogenesis of obesity. Only Ob PBMCs produce both IL-10 and IL-17 upon stimulation with PMA but this is not the case for H cells. However, in our test system, a key cytokine appears to be IL-21, as a product of various T cells, even including Th17 cells [39]. In fact, IL-21 acts as a strong inducer of Th17 cells, thus contributing to the maintenance of an inflammatory status in various pathologies [39, 40]. Quite interestingly, Ob PBMCs do not produce detectable amounts of IL-21 in the absence or presence of PMA. By contrast, H PBMCs spontaneously produce IL-21 and, to a higher extent, in the presence of PMA. In order to better clarify the present findings, one can hypothesize that Ob IL-10 keeps in check the overproduction of IL-17 in an indirect manner* via* abrogation of IL-21 production. However, our findings are in disagreement with those of current literature. In fact, Fabrizi et al. [13] demonstrated elevated expression of IL-21 and IL-21 mRNA in adipose tissue of high fat diet (HFD) mice and in the stromal vascular fraction of obese patients. In parallel, Treg cells were more abundant in IL-21 knockout mice in comparison to wild type animals fed both normal diet and HFD. These results have been confirmed by Knudsen and Lee [41] who demonstrated that IL-21 is a major negative regulator of interferon regulatory factor 4, thus inhibiting Treg cell accumulation in adipose tissue and systemic insulin sensitivity. Increase in IL-17 and IL-21/22 in the liver of HFD mice has been reported by Cavallari et al. [42]. However, these authors found elevated levels of the same cytokines in the ileum and colon of HFD mice. In a different clinical setting, recently we have reported an increase of IL-21 secretion from PBMCs of patients with chronic upper respiratory tract infections [43]. At the same time, an increase in IL-17 release was noted in the same patients, thus suggesting a positive correlation between IL-21 and IL-17 levels. It is likely that in our group of Ob people the maintenance of the inflammatory/anti-inflammatory equilibrium, as supported by IL-10 secretion in response to PMA, may lead to IL-21 suppression and contemporary containment of Th17 cell expansion.

Concerning proinflammatory cytokines, amounts of IL-1*β*, TNF-*α*, IL-6, and IL-8 are higher than those observed in the case of H individuals upon PMA stimulation, thus suggesting the propensity of Ob monocytes to polarize toward an inflammatory profile.

Addition of polyphenols to H and Ob cells does not affect absolute numbers of PBMCs and release of IL-4 and IL-17. Instead, polyphenols slightly increase IL-2 production in H cells at all concentrations but abrogate IL-2 release from Ob cells.

IL-10, as a major product of Treg cells [44], is significantly increased in H PBMCs at 5 *μ*g dose, as also previously reported [22]. In the case of Ob cells, the same dose leads to a trend to increase of this cytokine.

H cells in the presence of polyphenols increase the release of IL-21 in a dose-dependent fashion. The same event does not occur when Ob cells are treated with polyphenols, thus indicating that these compounds may act inhibiting the cellular sources of IL-21. In fact, there is evidence that dietary polyphenols are able to inhibit serum levels of IL-17 in volunteers who have ingested a high-caloric breakfast [45]. Finally, a significant decrease of IL-1*β* and IL-6 release can be observed when Ob PBMCs are treated with 5 *μ*g polyphenols in comparison to treated H cells with the same dose. This fact may be ascribed to the trend of polyphenol to increase IL-10 production by Ob cells.

## 5. Conclusions

With regard to polyphenol effects on Ob PBMCs, the most striking finding is represented by their inability to induce IL-21 production. On the other hand, in H individuals, polyphenols do increase production of IL-21, which contributes to the host protection, thus potentiating CD8 and natural killer cell function, and production of IgG1 by B cells [39]. In order to explain the polyphenol-mediated abrogation of IL-21 in Ob people, one should take into account the evidence that these compounds tend to increase the secretion of IL-10, which per se is enhanced under PMA stimulation.

From a translational point of view, our in vitro data suggest that assumption of dietary polyphenols by obese people may prevent obesity-related complications, even including vascular damage. This view is supported by a recent report which has demonstrated that pomegranate juice polyphenols are able to shift the M1 macrophage response to the M2 type response with production of IL-10 [46]. Therefore, this switch toward an anti-inflammatory macrophage phenotype has been shown to attenuate the inflammatory status of atherosclerotic aorta in mice. Additionally, one should also take into consideration the ability of polyphenols to reduce the reactive oxygen species (ROS) generation by human leukocytes [24]. In this respect, evidence has been provided that green tea polyphenol supplementation to obese women led to a decrease of ROS production by polymorphonuclear cells [47].

Taken together, all these findings support the usefulness of a dietary approach to limit inflammation in the course of obesity.

## Figures and Tables

**Table 1:** Main characteristic of Ob people.

Sex	Mean age	BMI (25.0–29.9) (overweight)	BMI (30.0–39.9) (moderate obesity)	BMI (>40.0) (severe obesity)
Female	44	5	4	3
Male	50	3	5	0
