# Corrigendum to “Immune Profile of Obese People and In Vitro Effects of Red Grape Polyphenols on Peripheral Blood Mononuclear Cells”

**DOI:** 10.1155/2017/4589705

**Published:** 2017-08-20

**Authors:** Thea Magrone, Emilio Jirillo, Anna Spagnoletta, Manrico Magrone, Matteo Antonio Russo, Sergio Fontana, Flavia Laforgia, Ilaria Donvito, Angelo Campanella, Franco Silvestris, Giovanni De Pergola

**Affiliations:** ^1^Department of Basic Medical Sciences, Neuroscience and Sensory Organs, University of Bari, 70124 Bari, Italy; ^2^Fondazione San Raffaele, 72013 Ceglie Messapica, Italy; ^3^MEBIC Consortium, San Raffaele Open University of Rome and IRCCS San Raffaele Pisana of Rome, Via di Val Cannuta, 247, 00166 Rome, Italy; ^4^Farmalabor Srl, 76012 Canosa di Puglia, Italy; ^5^Department of Biomedical Sciences and Human Oncology, Section of Clinical Oncology, University of Bari, 70124 Bari, Italy

In the article titled “Immune Profile of Obese People and In Vitro Effects of Red Grape Polyphenols on Peripheral Blood Mononuclear Cells” [1], there was an error in affiliation number three. The corrected affiliation is shown above.
